# Molecular Analysis of Prothrombotic Gene Variants in Patients with Acute Ischemic Stroke and with Transient Ischemic Attack

**DOI:** 10.3390/medicina57070723

**Published:** 2021-07-17

**Authors:** Gustavo Cernera, Marika Comegna, Monica Gelzo, Marcella Savoia, Dario Bruzzese, Mauro Mormile, Federica Zarrilli, Felice Amato, Pierpaolo Di Micco, Giuseppe Castaldo

**Affiliations:** 1 Dipartimento di Medicina Molecolare e Biotecnologie Mediche, Università di Napoli Federico II, 80131 Naples, Italy; gustavo.cernera@unina.it (G.C.); marika.comegna@unina.it (M.C.); monica.gelzo@unina.it (M.G.); marcella.savoia@unina.it (M.S.); felice.amato@unina.it (F.A.); giuseppe.castaldo@unina.it (G.C.); 2 CEINGE-Biotecnologie Avanzate, 80145 Naples, Italy; 3 Dipartimento di Sanità Pubblica, Università di Napoli Federico II, 80131 Naples, Italy; dario.bruzzese@unina.it; 4 Dipartimento di Medicina Clinica e Chirurgica, Università di Napoli Federico II, 80131 Naples, Italy; mormile@unina.it; 5 Dipartimento di Medicina Interna, Ospedale Fatebenefratelli, 80131 Naples, Italy; pdimicco@libero.it

**Keywords:** ischemic stroke, inherited thrombophilia, gene variants, genetic medicine, gene environmental interactions

## Abstract

*Background and objectives*: ischemic stroke (IS) is among the most frequent causes of death worldwide; thus, it is of paramount relevance to know predisposing factors that may help to identify and treat the high-risk subjects. *Materials and Methods*:we tested nine variants in genes involved in thrombotic pathway in 282 patients that experienced IS and 87 that had transient ischemic attacks (TIA) in comparison to 430 subjects from the general population (GP) of the same geographic area (southern Italy). We included cases of young and child IS to evaluate the eventual differences in the role of the analyzed variants. *Results*: we did not observe significant differences between TIA and the GP for any of the variants, while the allele frequencies of methylene-tetrahydrofolate reductase (MTHFR) C677T, beta-fibrinogen -455G>A and factor (FXIII) V34L were significantly higher in patients with IS than in the subjects from the GP. No significant interaction was observed with sex. *Conclusions*: the present data argue that some gene variants have a role in IS and this appears to be an interesting possibility to be pursued in large population studies to help design specific strategies for IS prevention.

## 1. Introduction

Ischemic stroke (IS) is among the most frequent causes of death and is the main cause of inability worldwide [1]. Although its rate increases with age, IS occurs in more than two million young adults (<45 years) per year worldwide, having an even more relevant clinical and socioeconomic impact on health care costs and loss of productivity [2]. Finally, even if it is rare, pediatric stroke is a leading cause of morbidity and mortality in children as well [3]; however, such genetic predisposition to thrombotic disease has been considered in several issues for pediatric patients, although not confirmed by further clinical studies. In 25% of cases, IS occurs after a history of transient ischemic attacks (TIA) [4]. Although there are differences between causes of IS in adult and in young populations [2], IS is considered a multifactorial disease that results from the co-existence of acquired and/or inherited predisposing factors that may interact [5,6,7]. In this complex pathophysiological mechanism, hypercoagulable states also are involved and may be associated to prothrombotic gene variants and/or antiphospholipid syndrome [8]. Historically, factor V Leiden (FVL) R506Q, prothrombin (FII) G20210A and methylene-tetrahydrofolate reductase (MTHFR) C677T variants with associated hyperhomocysteinemia are the most studied genetic factors in patients with IS. Moreover, other variants play a role in some forms of venous thrombosis including factor V R2 (FVR2) H1299R, factor XIII (FXIII) V34L, MTHFR A1298C, human platelet antigen (HPA)-1 L33P, beta fibrinogen -455G>A and plasminogen activator inhibitor (PAI)-I 4G/5G [5]. Conflicting results are present in the literature regarding the role of such variants as risk factors for ischemic stroke likely due to heterogeneous criteria used to select the patients in different studies or the fact that some studies compared the frequency of these gene variants in patients and controls from different geographical areas.

In this study, we aimed to test two prothrombotic and seven other gene variants in a large population of patients who experienced acute IS or TIA and compare them with the corresponding frequencies in subjects from the general population (GP) of the same geographic area (southern Italy).

## 2. Materials and Methods

### 2.1. Patients

The study was approved by the Ethical Committee of the University Federico II, Naples, Italy (protocol n. 370/18; 30 January 2019) and conducted in accordance with the Helsinki Declaration. All clinical and laboratory data were anonymized. Our laboratory acts as the reference lab for molecular diagnostics in the Campania region (southern Italy, about 5 million inhabitants). During the last twelve years (i.e., 2009–2020), we received thousands of requests for the molecular analysis of thrombophilia for different diseases [9,10,11,12]. For the present study, we retrospectively analyzed the results obtained in patients who experienced IS or TIA and compared them with subjects from the GP of the same geographical area (Campania region, southern Italy).

We recorded clinical data for each subject and verified that the diagnosis had been performed according to current guidelines [4,13]. Exclusion criteria: patients with a doubtful diagnosis. Clinical main neurological manifestations to suspect IS or TIA are summarized in Table 1. In addition, for cases in which multiple patients belonged to the same family, we considered the first family case analyzed for the gene variants panel. In detail, our study population included the following subjects:

(i) 87 patients who experienced transient ischemic attacks; median age: 52 years; range: 8–80 years; 48 females;
(ii) 282 patients who experienced at least one episode of acute ischemic stroke; median age: 52 years; range: 2–84 years; 146 females. Of these 282 patients, 21 (4.5%) had the first episode before 14 years (children stroke); median age: 7 years; range: 2–13 years; 7 females; while 71/282 patients (25.1%) had the first episode of stroke before 45 years (young stroke); median age: 39 years; range: 15–80; 48 females.

We compared the data to 430 subjects from the general population previously studied who, at the personal anamnesis (collected by a physician trained in the field), did not describe any episodes of venous nor arterial thrombosis (median age: 43 years; range: 5–85 years; 265 females); exclusion criteria: all cases in which a previous thromboembolic event was suspected [11,12].

As the objective of this was the allele frequency of many gene variants, we included in this retrospective analysis all cases of confirmed IS or TIA for any reason (i.e., atherothrombotic stroke or cardioembolic stroke); for the same reason, because the pathophysiological role of risk factors as smoking, diabetes, hypertension and dyslipidemia is a certainty, we retrospectively analyzed all subjects with IS or TIA independently from the presence of those predisposing risk factors.

### 2.2. DNA Extraction

Blood samples were collected by venipuncture into EDTA tubes using the Vacutainer system. DNA was extracted from leukocytes using a commercial automated procedure (Roche, Italy). The DNA was spectrophotometrically quantified (also to verify the purity) and analyzed for FVL (R506Q, rs6025); FVR2 (H1299R, rs1800595); FII (G20210A, rs1799963); MTHFR (C677T, rs1801133 and A1298C, rs1801131); beta-fibrinogen (-455 G>A, rs1800790); FXIII (V34L, rs5985); HPA-1 (L33P, rs5918) and PAI-1 (4G/5G alleles, rs1799889). All the variants were analyzed using a LightCycler 1.2 Instrument, which uses PCR for the amplification of the genomic region of interest and fluorogenic target-specific hybridization for the detection and genotyping of the amplified DNA, according to the manufacturer’s procedures (Roche Diagnostics). Finally, the results were analyzed using LightCycler Software 3.5. (Roche Diagnostics, Basel, Switzerland).

### 2.3. Statistical Analysis

Both allele and genotype frequencies are reported as absolute numbers and percentages. Differences among groups were accordingly assessed using the chi-square test or the Fisher exact test, when appropriate. Moreover, to quantify the effect of each variant on the disease risk, univariate odds ratios (ORs) with their corresponding 95% confidence intervals (95% CIs) were computed. Multivariable logistic regression models were finally built to assess whether gender could act as effect modifiers of the variant–disease association. In these models, for each polymorphism, the presence of disease was considered the dependent variable and the presence of the variant (in at least one allele) together with gender and their interaction entered as independent variable. The significance of the interaction coefficient should be interpreted as the presence of a different variant–disease association between males and females. All statistical analyses were conducted using the statistical platform R (version 4.0.1) [14].

## 3. Results

As shown in Table 2, we compared the allele frequency of the nine gene variants of the 87 patients with TIA and the 282 patients with IS with the GP group.

Interestingly, only PAI-I 4G/5G variant showed a significant difference in the allele frequency between patients with TIA and subjects of GP (*p* = 0.030) (Table 2); this association, however, was not confirmed when allele genotype was analyzed (Table 3).

On the other hand, the allele frequency of MTHFR C677T, beta-fibrinogen -455G>A and FXIII V34L was significantly higher in patients with IS than in GP (Table 2). This result depends on the different distribution of the genotype frequencies of these variants in the two different screened populations. In particular, we found that patients with IS showed an increased frequency of the following genotypes: (i) the TT genotype of the C677T MTHFR variant; (ii) the GA genotype of the beta-fibrinogen -455G>A variant; (iii) either the VL or the LL genotypes of the FXIII V34L variant (Table 3).

Accordingly, a significant increase in the odds of IS for each of these three variants was observed in subjects with variant allele (Table 4).

No significant interaction with gender was observed in the disease-variant association with respect to IS (Supplementary Figure S1), while a significant difference in the allele frequency distribution was only observed for the MTHFR C677T variant when classifying subjects with IS according to age at onset. In particular, patients with the first episode before 14 years of age were characterized by higher allele frequency with respect to subjects with a later onset (*p* = 0.026; Table 5).

## 4. Discussion

Ischemic stroke is one of the most common causes of disability and death in adults of Western countries, but the occurrence of IS is increasing in young people. Pathophysiology of IS is complex; several risk factors are involved, and the prothrombotic state is one of them. For this reason, besides common environmental risk factors such as smoking, hypertension, diabetes, obesity and dyslipidemia, inherited thrombophilia in the last decades has also been frequently investigated in patients with IS. Moreover, the frequent genetic predisposition of IS justifies the screening of inherited thrombophilia [7,8] in a complex model of gene and environmental causes.

Historically, hyperhomocysteinemia has been identified as a risk factor for IS in different studies and populations in particular when associated to the C677T MTHFR gene variant. However, although the occurrence of hyperhomocysteinemia is more frequent for the MTHFR C677T gene variant, A1298C MTHFR polymorphism has also been described as a risk factor for stroke in the Asian population and not in Europe [15,16]. Therefore, in our data, based on genetic testing, the increased frequency of MTHFR C677T could be expected while that of A1298C polymorphism would not. To support these data, our recent study concluded that the A1298C polymorphism was not associated with enhanced levels of serum homocysteine in our geographical area [17]. These results may play a role in the early identification of hyperhomocysteinemia and its genetic cause because its identification permits with folic acid supplementation a reduction of serum homocysteine levels that may induce a possible reduction of the number of clinical thrombotic events [18,19,20]. 

However, the daily clinical management of thrombotic diseases offers different pathogenetic mechanisms involved in venous thrombosis or arterial thrombosis and in this way also the presence of one or more gene variants that may predispose to hypercoagulable state may influence the clinical evolution to thrombosis in different ways.

Although such polymorphisms as FVL R506Q, FIIG20210A and MTHFR C677T seem to have a greater association toward venous thromboembolism, other genetic variants may also be associated with hypercoagulable states and then with thrombotic diseases. Among these, we studied in this report the frequency of the following variants: FVR2 H1299R that can cause an increased resistance to the cleavage by protein C together to FVL as far as the A1298C variant of MTHFR that can also impair the enzyme activity causing hyperhomocysteinemia as far as FXIII V34L (the L allele is associated with more efficient activation and stabilization of the coagulation system) and/or beta-fibrinogen -455G>A (this variant is associated with an alteration of the promoter region of the fibrinogen gene and can cause persistent elevated plasma fibrinogen levels) and/or PAI-1 4G/5G variants (the 4G allele is able to induce hypofibrinolysis) that with other related mechanisms have been described in selected population with arterial thrombosis, and HPA L33P is associated with hypercoagulation states, with consequent venous thrombotic complications. Of course, the genetic pattern is influenced by the presence of other environmental prothrombotic risk factors.

The current study showed that the allele frequency of the MTHFR C677T, FXIII V34L and beta-fibrinogen -455G>A variants was significantly higher in patients with IS as compared to the GP of the same ethnic-geographic area.

In our cohort of patients, C677T MTHFR gene polymorphism is the only gene variant that showed correlation with the onset of IS in young age. In this way, the role of prevention is intriguing because lowering homocysteine levels in young people could reduce the number of clinical events, improving the outcome of affected patients and reducing morbidity for neurological disease in young people.

Moreover, the statistical association of FXIII V34L and beta-fibrinogen -455G>A in our population of patients with IS compared with data available from GP patients could be considered unexpected and interesting. Previous studies that reviewed the chance of this polymorphism to be a risk factor for IS did not find a strong association. However, most studies analyzed low number of patients, often less than 100 [21]. Therefore, based on the low number of patients reported in previous studies, we could consider our results, which associated the V34L variant of FXIII in patients with IS, of particular interest and deserving to be considered for evaluation in an enlarged population of patients with IS of the same geographical area.

The beta-fibrinogen -455G>A variant has been found with a higher frequency among patients with IS and acute myocardial infarction and it has been considered as a risk factor for IS among Asians [22]. In this way, our data are in agreement and could give the suggestion to routinely test plasma levels of fibrinogen in patients with IS [23] as far as the follow up for the secondary stroke prevention.

Other gene variants that were analyzed in our study did not find a statistical correlation with the occurrence of IS or TIA. These results may raise the daily clinical dilemma about the role of inherited thrombophilia in cerebrovascular diseases in particular for the possible causal or coincidental role in pathophysiology of cerebral ischemia; however, in this clinical setting, the role of inherited thrombophilia may be considered fundamental because it may be involved in recurrent thrombosis that usually affects carriers of those inherited clotting abnormalities. 

For all examined gene variants, no gender differences were found for either TIA or IS, suggesting that the genetic predisposition does not have a relationship with gender. These data show a small difference with those reported in other studies regarding other atherothrombotic diseases such as AMI [10,11,12] and so further analysis are suggested. 

A further clinical aspect that we noted was the low clinical incidence of other thrombotic diseases in first-degree relatives of the studied patients (e.g., venous thromboembolism or thrombotic stroke); of course, this aspect may be related also to the presence of different environmental thrombotic risk factors that usually influence the occurrence of venous thromboembolism.

Summarizing our results, we suggest that the analyzed gene variants have a minor role as risk factors for TIA, but these data need a further clinical and genetic interpretation because we should consider that up to 25% of IS appear in subjects that previously experienced TIA [4]. Then, although not with a clear statistical association, the question on routine testing for these variants involved in the thrombotic pathway in patients with TIA remains open, in particular for the primary prevention of IS. We should consider, in fact, that our data reported that there is a relationship between IS and three gene variants and one of these showed a relationship also between the early onset of IS at young age. The role of genetic testing is not only useful to explain pathophysiological mechanisms but also to establish an effective secondary prevention of affected patients and/or a primary prevention of patients with similar risk factor and from the same geographical area. Interestingly, some of the analyzed genetic variants showed no significant association differently from those found in other selected population with IS already reported in the literature [24] confirming that the complex gene–environmental and/or gene–gene interaction and the multifactorial genesis of IS need to be furtherly investigated. Therefore, in the last years, some studies on populations from several geographical areas have highlighted the role of other loci involved in the pathophysiology of ischemic stroke [25,26].

In the TIA group, non-genetic risk factors could have a determining role due to the transient nature of the event; from a clinical point of view, this finding may be associated to a larger list of inclusion/exclusion criteria for TIA compared to those for IS [27]. Moreover, the study limitations of our report may be considered: small sample size, especially when trying to explore interaction with gender through subgroup analyses; and the difficult evaluation of other risk factors for IS or TIA besides age and gender due to retrospective analysis.

## 5. Conclusions

We could conclude that the present data argue that some of these gene variants involved in the thrombotic pathway have a role in IS in this geographic area and this could lead to further studies that should consider a larger population and thorough evaluation of these and other genetic variants with environmental risk factors for stroke to plan specific strategies for primary and secondary IS prevention. 

## Figures and Tables

**Table 1:** Frequency of main neurological symptoms, present for more than 24 h in ischemic stroke (IS, *n* = 282) and present for less than 24 h in transient ischemic attack (TIA, *n* = 87).

	IS	TIA	*p* *
Unilateral paresis	98 (34.8)	23 (26.4)	0.189
Mental disorders and confusion	58 (20.2)	25 (28.7)	0.148
Hemianopia/diplopia	10 (3.5)	2 (2.3)	0.739
Dysartria	76 (27.0)	23 (26.4)	1.000
Headache	25 (8.9)	10 (11.5)	0.601
Blurred vision	15 (5.3)	4 (4.6)	1.000

* *p*-value by chi-square/Fisher test.

**Table 2:** Allele frequency, *n* and (%) of the nine gene variants. Comparison between ischemic stroke (IS, *n* = 282) or transient ischemic attack (TIA, *n* = 87) versus the general population (GP, *n* = 430).

	GP	IS	*p* *	TIA	*p* **
FV R506Q (FVL)	21 (2.4)	22 (3.9)	0.153	2 (1.2)	0.404
FV H1299R (FVR2)	41 (5.0)	28 (5.0)	1	10 (5.6)	0.839
FII G20210A	23 (2.7)	23 (4.1)	0.194	8 (4.6)	0.273
MTHFR C677T	376 (43.8)	292 (52.1)	0.003	83 (47.7)	0.393
MTHFR A1298C	249 (29.0)	159 (28.3)	0.834	49 (28.2)	0.905
beta-fibrinogen -455G>A	171 (19.9)	145 (26.5)	0.005	43 (24.7)	0.183
FXIII V34L	153 (17.8)	141 (25.5)	0.001	34 (19.5)	0.661
HPA L33P	128 (14.9)	85 (15.3)	0.895	28 (16.2)	0.727
PAI-I 4G/5G	399 (46.4)	257 (46.1)	0.944	97 (55.8)	0.030

* *p*-values for comparison between IS and GP population; ** *p*-values for comparison between TIA and GP population.

**Table 3:** Genotype frequency, *n* and (%) of the nine gene variants. Comparison between ischemic stroke (IS, *n* = 282) or transient ischemic attack (TIA, *n* = 87) versus the general population (GP, *n* = 430).

	GP	IS	*p* *	TIA	*p* **
FV R506Q (FVL)			0.147		0.398
RR	409 (95.1)	259 (92.2)		85 (97.7)	
QR	21 (4.9)	22 (7.8)		2 (2.3)	
QQ	0	0		0	
FV H1299R (FVR2)			0.649		0.691
HH	369 (90.4)	254 (90.1)		77 (88.5)	
HR	37 (9.1)	28 (9.9)		10 (11.5)	
RR	2 (0.5)	0		0	
FII G20210A			0.186		0.266
GG	404 (94.6)	258 (91.8)		79 (90.8)	
GA	23 (5.4)	23 (8.2)		8 (9.2)	
AA	0	0		0	
MTHFR C677T			0.01		0.435
CC	138 (32.2)	64 (22.9)		22 (25.3)	
CT	206 (48.0)	140 (50.0)		47 (54.0)	
TT	85 (19.8)	76 (27.1)		18 (20.7)	
MTHFR A1298C			0.687		0.977
AA	222 (51.6)	144 (51.2)		46 (52.9)	
AC	167 (38.8)	115 (40.9)		33 (37.9)	
CC	41 (9.5)	22 (7.8)		8 (9.2)	
beta-fibrinogen -455G>A			0.002		0.277
GG	273 (63.5)	138 (50.4)		48 (55.2)	
GA	143 (33.3)	127 (46.4)		35 (40.2)	
AA	14 (3.3)	9 (3.3)		4 (4.6)	
FXIII V34L			0.003		0.711
VV	291 (67.7)	156 (56.3)		57 (65.5)	
VL	125 (29.1)	101 (36.5)		26 (29.9)	
LL	14 (3.3)	20 (7.2)		4 (4.6)	
HPA L33P			0.965		0.077
LL	313 (72.8)	201 (72.3)		64 (74.4)	
LP	106 (24.7)	69 (24.8)		16 (18.6)	
PP	11 (2.6)	8 (2.9)		6 (7.0)	
PAI-I 4G/5G			0.992		0.07
4G/4G	125 (29.1)	82 (29.4)		16 (18.4)	
4G/5G	211 (49.1)	137 (49.1)		45 (51.7)	
5G/5G	94 (21.8)	60 (21.5)		26 (29.9)	

* *p*-values for comparison between IS and GP population; ** *p*-values for comparison between TIA and GP population.

**Table 4:** Odds ratios (ORs) with the corresponding 95% confidence intervals (95% CIs) of some gene variants for ischemic stroke (IS *n* = 282) (GP, *n* = 430).

	GP	IS	OR (95% CI)	*p* Value
MTHFR (C677T)	291 (67.8)	216 (77.1)	1.6 (1.13–2.26)	0.007
FXIII (V34L)	139 (32.2)	121 (43.7)	1.62 (1.19–2.22)	0.002
beta-fibrinogen -455G>A	157 (36.5)	136 (49.6)	1.71 (1.26–2.33)	0.001

**Table 5:** Allele frequency, *n* and (%) of nine variants in different age groups with ischemic stroke.

	Children (≤14 ys)	Young (15–44 ys)	Adult (≥45)	*p*-Value
FV G1691R (FVL)	2/42 (4.8)	3/142 (2.1)	17/378 (4.5)	0.428
FV H1299R (FVR2)	0/42 (0.0)	5/142 (3.5)	23/380 (6.1)	0.185
FII G20210A	1/42 (2.4)	7/140 (5.0)	15/380 (3.9)	0.774
MTHFR C677T	26/42 (61.9)	60/140 (42.8)	206/378 (54.5)	0.026
MTHFR A1298C	10/42 (23.8)	39/142 (27.5)	110/378 (29.1)	0.746
beta-fibrinogen -455G>A	11/42 (26.2)	41/138 (29.7)	93/370 (25.1)	0.581
FXIII V34L	14/40 (35.0)	39/142 (27.5)	88/372 (23.6)	0.240
HPA L33P	8/42 (19.0)	23/138 (16.7)	54/379 (14.2)	0.613
PAI-I 4G/5G	18/42 (42.9)	76/140 (54.3)	163/378 (43.1)	0.071

## Data Availability

Data sharing is not applicable to this article.

## Supplementary Materials

The following are available online at https://www.mdpi.com/article/10.3390/medicina57070723/s1.
