# Supplementary figures and images for: Molecular Analysis of Prothrombotic Gene Variants in Patients with Acute Ischemic Stroke and with Transient Ischemic Attack
Source: Medicina (Kaunas). 2021 Jul 17;57(7):723. doi: 10.3390/medicina57070723 (PMC8306646; doi:10.3390/medicina57070723)

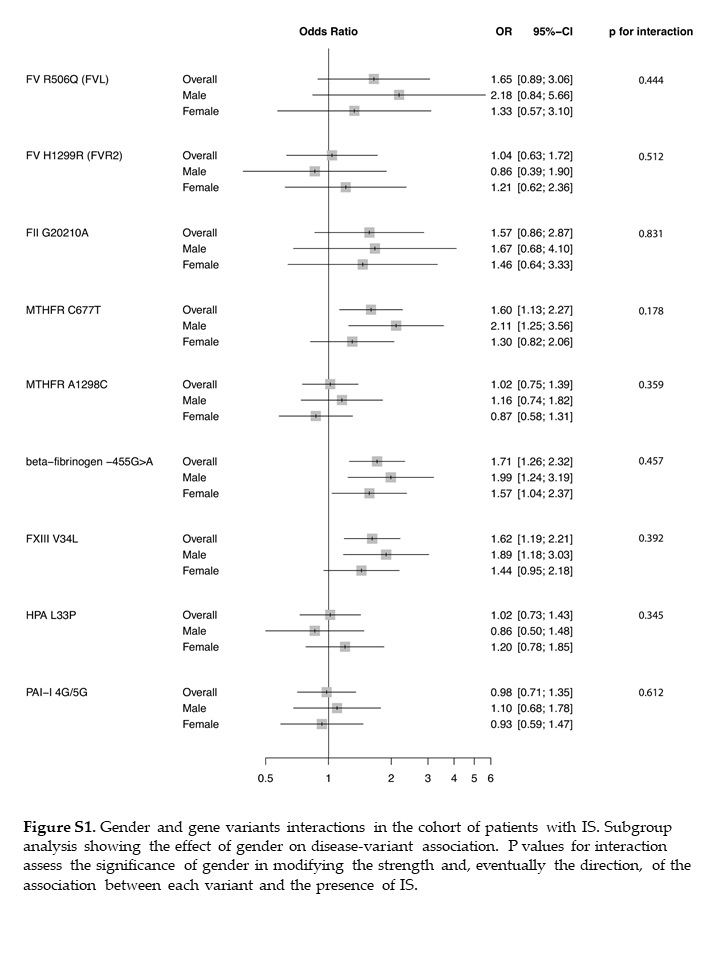

Supplement: Supplementary file 1 [file medicina-57-00723-s001.zip › medicina-1252076-supplementary.tif]
